# Factors Associated With Suicide Among Patients With Lung Cancer in the United States: A Retrospective Cohort Study Based on Surveillance, Epidemiology, and End Results (SEER) Data

**DOI:** 10.7759/cureus.77298

**Published:** 2025-01-11

**Authors:** Praneet Iyer, Anjana Delhi, Omar H Fahmy, Esha Sharma

**Affiliations:** 1 Internal Medicine/Pulmonary and Critical Care, Louisiana State University Health Sciences Center, Shreveport, USA; 2 Cardiovascular Surgery, Washington University in St. Louis, St. Louis, USA; 3 Family Medicine, MultiCare Good Samaritan Hospital, Puyallup, USA; 4 Family Medicine, Louisiana State University Health Sciences Center, Monroe, USA

**Keywords:** lung cancer, risk factors, suicide, surveillance epidemiology and end results (seer), united states

## Abstract

Background

There are significantly higher suicide rates among patients with lung cancer vs. the general population, as well as individuals with other cancer types. This study was conducted to determine the factors associated with suicide among patients with lung cancer.

Methods

A total of 1,007,088 lung cancer patients diagnosed between 2000 and 2020 from the Surveillance, Epidemiology, and End Results (SEER) database were selected for the study. Chi-square and t-test were used for descriptive analysis of categorical and continuous variables, respectively. Kaplan-Meier plot, log-rank test, and Cox proportional hazard test were used for survival analysis. The study cohort after the exclusions consisted of 843,750 participants.

Results

Among 843,750 lung cancer patients included in this study, suicide was the cause of death in 1,014 (0.12%) patients. The overall mean survival in subjects who committed suicide was 26 months. Higher suicide rates were observed in the elderly, male sex, White race, distant lung cancer, and patients dwelling in metropolitan areas. After adjusting, patients with distant-stage lung cancer had a 1.6 times higher hazard of suicide compared to stage localized cancer patients with a hazard ratio of 1.59 (1.32, 1.90, p < 0.001).

Conclusion

A lung cancer diagnosis is associated with a higher risk of suicide compared to the general population and patients with other cancer types. We suggest that screening for distress, using the National Comprehensive Cancer Network (NCCN) distress screening tool, and depression at regular intervals in patients with lung cancer is imperative to mitigate the non-cancer loss of life.

## Introduction

Lung cancer is the third most common malignancy in the US and the second most common globally in both men and women [1,2]. In the US, lung cancer by far accounts for the highest cancer-related mortality. According to the cancer statistics provided by the Centers for Disease Control and Prevention (CDC), 46.4% of the patients had distant disease at the time of diagnosis, and 48.9% had localized or regional disease. The estimated five-year relative survival rate among patients with lung cancer in the US is 24% [3]. The American Cancer Society has projected approximately 238,000 new lung cancer diagnoses in the US in 2023 and nearly 127,000 deaths from lung cancer [1].

A diagnosis of lung cancer is highly complex and overwhelming, and individuals who receive this diagnosis undergo profound levels of distress and anxiety that can be devastating. The rates of depression and anxiety disorders, solitary or intermixed, in patients with lung cancer surpass those associated with most other cancer types, which stem from the awareness of survival rates and implications of the therapy on overall health and uncertainty about the future. Lung cancer patients can undergo a dramatic change of losing functionality and eventually becoming dependent. Social, demographic, and disease factors are major determinants that collectively influence the magnitude of physical and emotional suffering among those patients [4].

Population-based studies scrutinizing suicidality in patients with cancer reveal significantly higher suicide rates among patients with lung cancer compared to the general population as well as individuals with other cancer types [5-14]. The observations are consistent across studies conducted in the US and other European and non-European countries. Persons with lung cancer not only have a high suicide risk, but they also pursue medically assisted suicide or death services at higher rates compared to other cancers. In analyses of assisted termination of life in cancer patients, lung cancer accounted for 14% to 17.6% of all cases [15,16].

This study aims to determine the risks and predictors of suicide among patients with lung cancer in the US based on data from the National Cancer Institute Surveillance, Epidemiology, and End Results (SEER) database.

## Materials and methods

A total of 1,007,088 lung cancer patients were obtained from the SEER database, released on 8/14/23, consisting of 17 US registries (covering 26.5% of the US population) from 2000 to 2020. SEER*Stat software 8.4.2 (Surveillance Research Program at the National Cancer Institute, Bethesda, MD) was used to extract the data. Participants with ages less than 18 years were excluded from the study, and complete cases were only included. The study cohort after the exclusions consisted of 843,750 participants.

Variables

The variables collected for each patient included age at diagnosis (age was recoded to below 50 years and 50 and above), race (race and origin recode variable in the SEER data includes Hispanic, non-Hispanic White, non-Hispanic Black, non-Hispanic Asian/Pacific Islander (API), non-Hispanic American Indian/Alaska Native (AI/AN), non-Hispanic unknown race was reclassified as White, Black, Hispanic, and other race), gender, year of diagnosis of lung cancer, cause of death (COD to site recode), which was reclassified as a binary variable with suicide and self-inflicted injury (event) and all other causes of death (no event), summary stage 2000 (1998-2017), months from diagnosis to treatment, survival months (time in months from diagnosis to death or censoring), marital status at diagnosis which includes categories divorced, married, separated, single (never married), unmarried or domestic partner, widowed was reclassified into four groups (married or with partner, divorced or widowed or separated, single, and unknown). Median household income adjusted for inflation in 2021, which was recategorized into three groups: <50,000, 50,000-74,999, and 75,000 and above. Rural-to-urban continuum code, which was recategorized into three groups, including metropolitan, non-metropolitan adjacent to metropolitan, and rural. We decided to use the summary stage variable 2000 (1998-2017), which classified the stages as localized, regional, unknown or unstaged, and distant, as it had the most complete information. Suicide and self-inflicted injury as a COD was used as the event or outcome.

The study design is a retrospective cohort study. Permission was obtained from SEER to use the data, and IRB consent was not required since only publicly available deidentified data was used. R version 4.1.3 (R Foundation for Statistical Computing, Vienna, Austria) was used for statistical analysis.

## Results

Baseline characteristics

Chi-square and t-test were used for descriptive analysis of categorical and continuous variables, respectively. A total of 843,750 lung cancer patients were included in this study; 36,745 (4.4%) cases were below the age of 50, and 807,005 (95.6%) were 50 and above. Stage distant included 447,385 cases (53%), stage localized included 158,379 cases (18.8%), regional included 190,591 cases (22.6%), and unknown and unstaged included 47,395 cases (6.6%). The males were 448,693 (53.2%), and females were 395,057 (46.8%). Race consisted of White (658,983 (78.1%)), Black (84,323 (10%)), Hispanic (47,671 (5.6%)), and others (52,773 (6.3%)). The income groups consisted of <$50,000 (113,471 (13.4%)), $50,000-$74,999 (423,463 (50.2%)), and >$75,000 (306,816 (36.4%)). The rural-urban continuum consisted of metropolitan (707,690 (83.9%)), adj metropolitan (79,053 (9.4%)), and rural (57,007 (6.8%)). Marital status consisted of partner/married (425,681 (50.5%)), divorced/widowed/separated (277,160 (32.8%)), single (104,189 (12.3%)), and unknown (36,720 (4.4%)).

The baseline demographic characteristics of the patients who had the event (suicide) and those who did not have the event are presented in Table 1. Stage, sex, race, and rural/urban were significantly different among the lung cancer patients with the event (suicide) compared to those without the event.

**Table 1:** Baseline demographic characters of the patients who had the event (suicide) and those who did not have the event Adj: adjacent; div: divorced; sep: separated; wid: widowed

Variable	No event	Event	p-value
	n = 842,736	n = 1,014	-
Survival month	26.0 (40.0)	24.9 (37.4)	0.37
Age			
Below 50 years	36,710 (4.4%)	35 (3.5%)	0.182
50 years and above	806,026 (95.6%)	979 (96.5%)	-
Stage			
Distant	446,926 (53%)	459 (45.3%)	<0.001
Localized	158,137 (18.8%)	242 (23.9%)	-
Regional	190,326 (22.6%)	265 (26.1%)	-
Unknown/unstaged	47,347 (5.6%)	48 (4.7%)	-
Sex			
Female	394,915 (46.9%)	142 (14%)	<0.001
Male	447,821 (53.1%)	872 (86%)	-
Race			
White	658,071 (78.1%)	912 (89.9%)	<0.001
Black	84,299 (10%)	24 (2.4%)	-
Hispanic	47,640 (5.7%)	31 (3.1%)	-
Other	52,726 (6.3%)	47 (4.6%)	-
Income			
<50,000	113,317 (13.4%)	154 (15.2%)	0.027
$50,000-$74,999	422,933 (50.2%)	530 (52.3%)	-
>$75,000	306,486 (36.4%)	330 (32.5%)	-
Urban/rural			
Metropolitan	706,881 (83.9%)	809 (79.8%)	<0.001
Adj metropolitan	78,923 (9.4%)	130 (12.8%)	-
Rural	56,932 (6.8%)	75 (7.4%)	
Marital status			
Partner/married	425,167 (50.5%)	514 (50.7%)	0.064
Div/wid/sep	276,856 (32.9%)	304 (30%)	-
Single	104,040 (12.3%)	149 (14.7%)	-
Unknown	36,673 (4.4%)	47 (4.6%)	-

Survival analysis: summary stage

**Figure 1:**
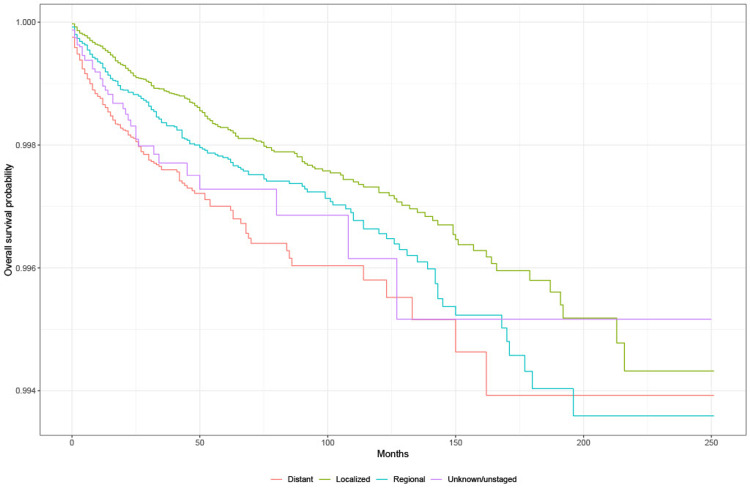
Survival by lung cancer staging

Kaplan-Meier (KM) survival analysis showed that the probability of survival from suicide is highest among unknown/unstaged group lung cancer patients compared to other stages. Based on the log-rank test, there is a significant difference in the probability of survival between stage distant and localized and stage distant and regional (p < 0.05) (Figure 1).

Survival analysis: sex

**Figure 2:**
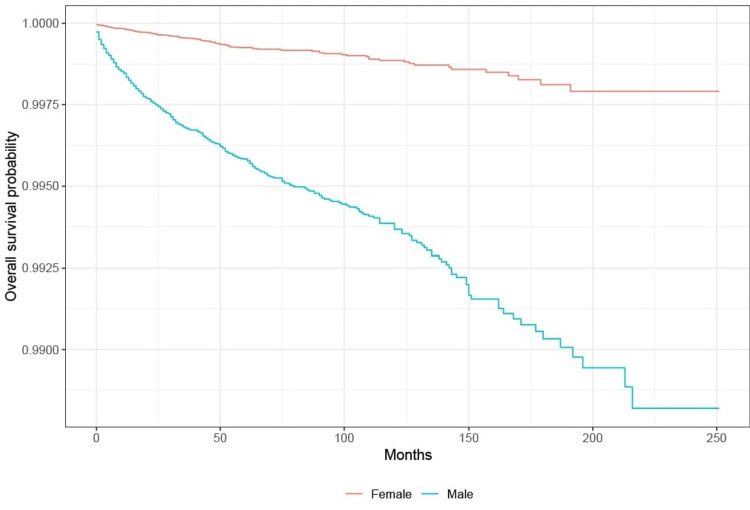
Survival by gender in lung cancer patients

KM survival analysis showed that the probability of survival from suicide is highest among females with lung cancer compared to males with lung cancer. Based on the log-rank test, there is a significant difference in the probability of survival from suicide between males and females with lung cancer (p < 0.001) (Figure 2).

Survival analysis: race

**Figure 3:**
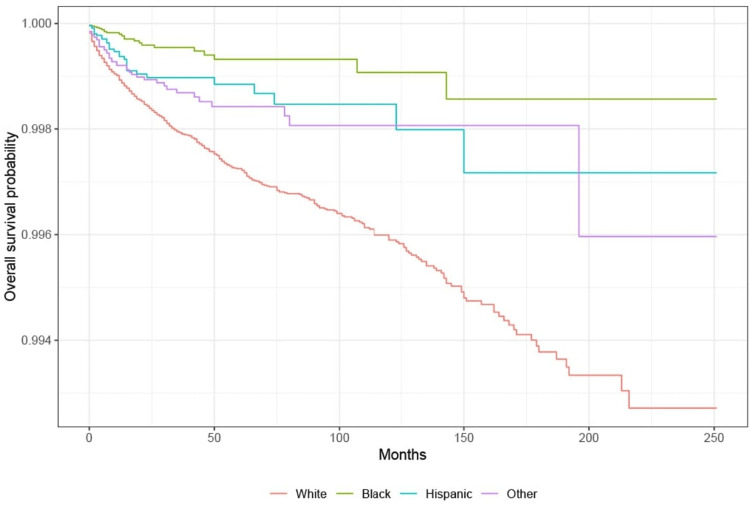
Survival based on race in lung cancer patients

KM survival analysis showed that the probability of survival from suicide is highest among those belonging to the Black race. The lowest survival rate was for those belonging to the White race. Based on the log-rank test, there is a significant difference in the probability of survival from suicide in all groups with lung cancer (p < 0.001) except other races and Hispanic (Figure 3).

Survival analysis: income

**Figure 4:**
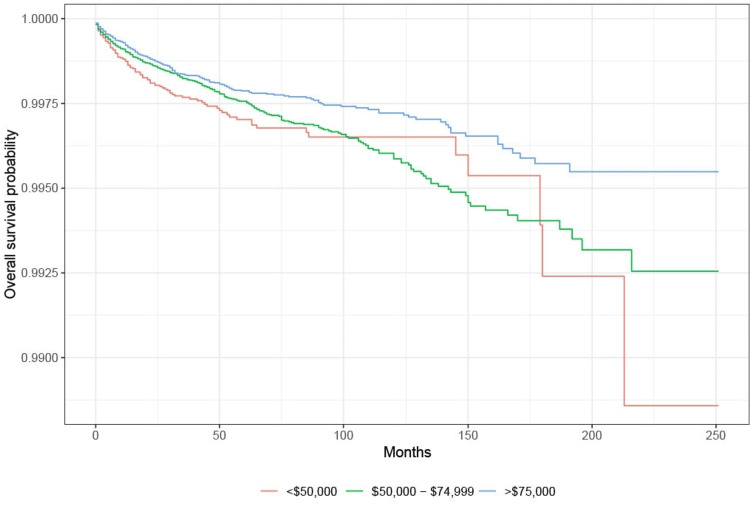
Survival based on income in lung cancer patients

KM survival analysis showed that the probability of survival from suicide is highest among those with >75,000 income compared to those with lower income groups. Based on the log-rank test, there is a significant difference in the probability of survival from suicide among those with income >$75,000 (50,000-74,999), as well as <50,000 (p < 0.001), but between the groups of 50,000-74,999 and <50,000, there is no significant difference in survival (p < 0.07) (Figure 4).

Survival analysis: rural-urban continuum

**Figure 5:**
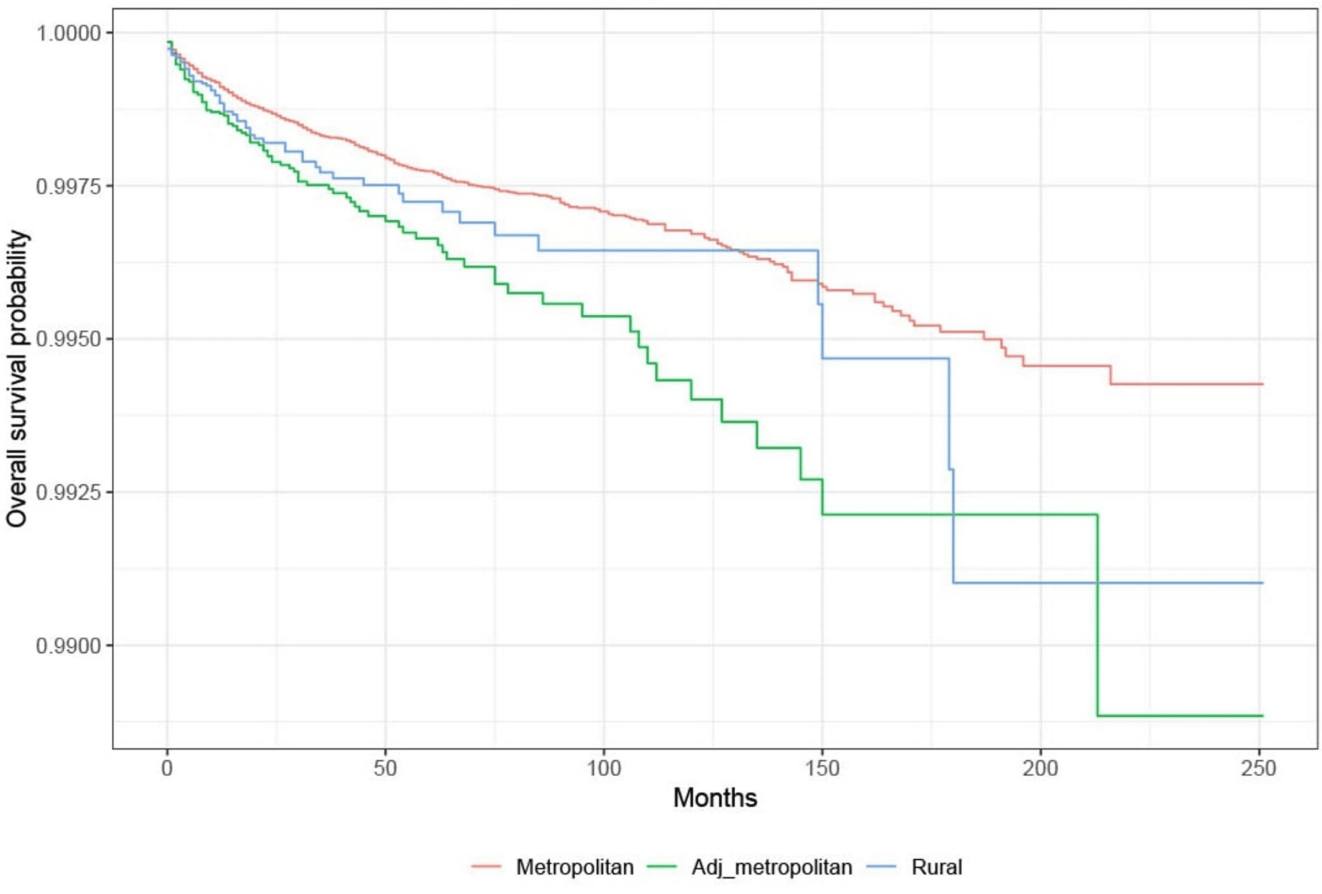
Survival based on rural-urban continuum in lung cancer patients Adj: adjacent

KM survival analysis showed that the probability of survival from suicide is highest among those in metropolitan areas compared to other groups. Based on the log-rank test, there is a significant difference in the probability of survival from suicide among those living in metropolitan areas compared with those living in areas adjacent to metropolitan areas (p < 0.001). There is no difference between rural areas and the other two groups (p > 0.05) (Figure 5).

Survival analysis: marital status

**Figure 6:**
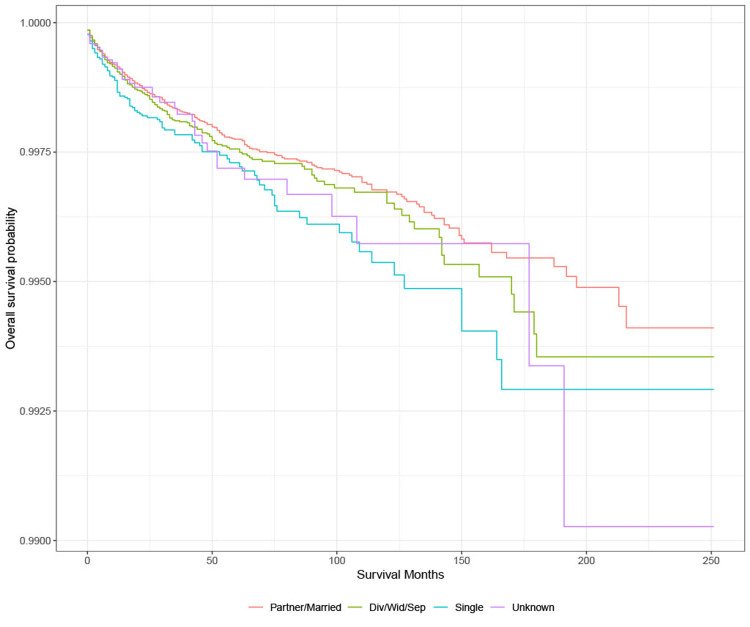
Survival based on marital status in lung cancer patients Div: divorced; sep: separated; wid: widowed

KM survival analysis showed that the probability of survival from suicide is highest among those who are divorced, separated, or widowed group. Based on the log-rank test, there is no significant difference in the probability of survival from suicide among those with different marital status groups with lung cancer (Figure 6).

Survival analysis: age

**Figure 7:**
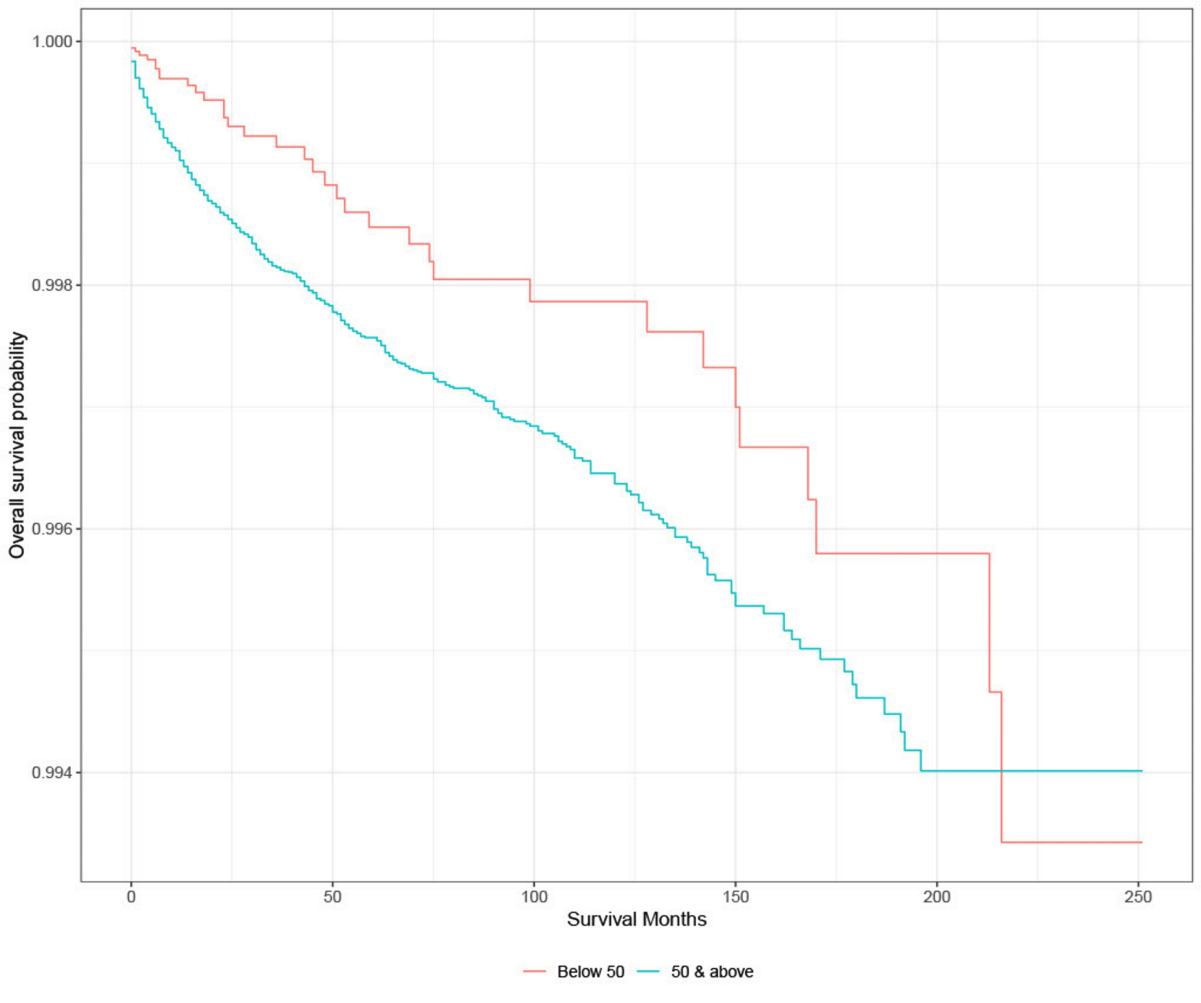
Survival based on age in lung cancer patients

KM survival analysis showed that the probability of survival from suicide is highest among those aged 50 and above lung cancer patients compared to those below 50. Based on the log-rank test, there is no significant difference in the probability of survival from suicide between age 50 and above and those below age 50 (p > 0.05) (Figure 7).

Cox proportional model

The Cox proportional model (Table 2) was run adjusted for age group, race, gender, and rural-urban status. After adjusting for sex, race, and rural-urban status, patients with distant stage lung cancer had a 2.1 higher hazard of suicide compared to stage localized cancer patients (HR = 2.1, 95% CI (1.78, 2.48), p < 0.001).

**Table 2:** Adjusted Cox proportional model of predictors of suicide in lung cancer patients

Characteristic	Suicide		p-value
	Hazard ratio	95% CI	-
Summary stage			
Localized	Reference	-	-
Regional	1.23	1.03, 1.46	0.02
Distant	2.10	1.78, 2.48	<0.001
Unknown	1.61	1.18, 2.20	0.002
Income			
<50,000	Reference	-	-
$50,000-74,000	0.95	0.77, 1.17	0.611
>$75,000	0.77	0.61, 0.98	0.031
Race			
White	Reference	-	-
Black	0.19	0.13, 0.29	<0.001
Hispanic	0.47	0.33, 0.67	<0.001
Other	0.57	0.43, 0.77	<0.001
Urban-rural			
Metropolitan	Reference	-	-
Adjacent to metropolitan	1.20	0.97, 1.49	0.08
Rural	0.94	0.72, 1.24	0.68
Sex			
Female	Reference	-	-
Male	6.52	5.46, 7.79	<0.001

In the adjusted model, the hazard of suicide was 0.77 times lower in people with income of >75,000 as compared to those with income of <50,000 (HR 0.77, 95% CI (0.61,0.0 = 98), p < 0.05). In the adjusted model, the hazard of suicide was 0.19 times lower among those belonging to the Black race compared to the White race (HR = 0.19, 95% CI (0.13, 0.29), p < 0.001). In the adjusted model, the hazard of suicide was 0.47 times lower among those belonging to Hispanic origin compared to White (HR = 0.47, 95% CI (0.33, 0.67), p < 0.001). In the adjusted model, the hazard of suicide was 0.75 times lower among those belonging to another race compared to Whites (HR = 0.75, 95% CI (0.43, 0.77), p < 0.001). In the adjusted model, the hazard of suicide was 6.52 times higher among men than women (HR = 6.52, 95% CI (5.46, 7.79), p < 0.001). In the adjusted model, the hazard of suicide was 1.2 times higher among those living adjacent to metropolitan areas than those living in metropolitan areas.

## Discussion

This study indicates that the risk of suicide was highest among patients who were of male gender (HR 6.52), White race (Black HR 0.19, Hispanic HR 0.47, and other races HR 0.75), with distant lung cancer (HR 2.1), and who lived in places adjacent to metropolitan areas (HR 1.2). Multiple studies have shown an increased risk of suicide in the older population due to lower quality of life, more comorbidities, and poor response to treatment for lung cancer, but this was not the case in our study. Males had a higher risk than females as they were seen to mostly live alone and not rely on guidance from their family, which led to a lower social support system. Most of the studies consistently observed White race to have an increased risk for suicide as compared to other races, except one study in which Asians were more likely to commit suicide [7,8,11-13,15-19,20,21]. The reasons for the White race to have an increased risk of suicide were mostly unclear except in one study, where it was seen that suicide rates were lower in immigrant-rich neighborhoods. This was because they lived together in larger groups, which provided a stronger social support network, and the cultural fabric of foreign-born patients made suicide a very unpopular idea and essentially a taboo. These factors were seen as lacking in patients belonging to the white race, who mostly lived in isolated families [10,13,16,17,20].

Previous studies have also shown distant or advanced lung cancers to have a higher risk of suicide. These patients have a higher incidence of emotional distress, anxiety, and depression, which was not recognized until very late in the course of the disease [8,9,11,12]. This was also the case in separated and divorced couples who went through more emotional distress and anxiety without a good social support system, due to which they were more likely to die by suicide. In addition, they were noted to have more issues with shortness of breath, swallowing difficulties, and cancer-related pain, thereby reducing their quality of life [14-16]. These patients were seen to get non-curative or palliative treatment, which increased their sense of hopelessness, thus adding to the risk of death by suicide [18-23]. Mostly, patients living in areas with lower education levels, lower socioeconomic status, and higher unemployment were seen to have higher rates of suicide. Lower education level led to a lack of understanding of the disease process and prognosis, resulting in more distress and a higher risk of suicide. Also, due to the high costs of the treatments for lung cancer and lack of availability of palliative care as well as a psychiatric referral system in underserved sections of society and the ones with higher unemployment rates, patients lacked the means to obtain treatment and get professional assistance for depression and other forms psychiatric illnesses [10,11,19]. Due to all the factors mentioned above, these patients were noted to be at higher risk of suicide, too, which aligns with the prior literature review.

In addition, patients living in metropolitan areas were seen to have higher survival rates in our study as compared to patients living in areas adjacent to metropolitan areas and rural areas. Prior studies showed that patients living in areas adjacent to metropolitan areas and rural areas had lower educational status, lived in poverty, and were unemployed. Suicide rates were higher among marginalized cancer patients due to poor socioeconomic conditions and poor social support systems. The paucity of jobs in rural and areas adjacent to metropolitan areas was associated with economic uncertainty and issues with the affordability of health care, which led to increased suicides [10].

Strengths

The sample size is large. The SEER dataset is representative of the US population and can be generalized. The study design is a retrospective cohort study. It fills the gap in the current literature about the association between the stage of lung cancer and suicide, as data from 2000 to 2020 were included in the study. As per our literature search, there have not been any studies published so far that included recent SEER data until 2023.

Limitations

Stages of lung cancer were not available for all cases. In this current dataset, depression or anxiety or emotional distress, education status, time interval from diagnosis to suicide, chemotherapy/radiation treatment, insurance status, and tobacco/alcohol variables were not available and, therefore, were not included in the analysis.

## Conclusions

We believe that the results of our study would be useful for different specialties like oncology, pulmonology, psychiatry, and primary care to understand the non-oncological aspects of lung cancer. We hope that this study may assist in promoting collaboration between different healthcare providers to assist the patients with holistic care, not only focused on treating cancer but also, and importantly, addressing the social, emotional, physical, and other medical needs of the patients to mitigate the suicidality and potentially the thoughts of hurting oneself. In addition, the results may help in the identification of lung cancer patients who are at higher risk of committing suicide so that appropriate interventions, including psychiatric referral, psychotherapy, palliative care evaluation, etc., can be applied to them at appropriate time intervals after diagnosis to decrease any unnecessary loss of life.
